# Supplementary material for: Comprehensive fitness maps of Hsp90 show widespread environmental dependence
Source: eLife. 2020 Mar 4;9:e53810. doi: 10.7554/eLife.53810 (PMC7069724; doi:10.7554/eLife.53810)
Supplement: Figure 6—figure supplement 2—source data 1. [file elife-53810-fig6-figsupp2-data1.docx]

**Figure 6 – figure supplement 2, source data.** The Spearman’s rank correlation coefficients (r) and associated p-values between molecular features and selection coefficients for each environment.

| **Condition** | **Feature** | **Correlation** | **P-value** |
| --- | --- | --- | --- |
| Standard | Distance from ATP | 0.4527 | 4.25E-32 |
| Standard | SASA by position | 0.3564 | 2.72E-19 |
| Standard | ΔΔG by mutation | -0.1715 | 6.88E-80 |
| Standard | Δ Hydrophobicity by mutation | 0.1170 | 1.30E-43 |
| Standard | Δ Flexibility by mutation | 0.0972 | 1.43E-30 |
| Standard | Δ pI by mutation | -0.0340 | 6.00E-05 |
| Standard | Δ log(Solubility) by mutation | -0.0203 | 0.0168 |
| Standard | Δ Volume by mutation | -0.0225 | 0.0081 |
| Standard | Δ P(helix by mutation) | -0.0216 | 0.0110 |
| Standard | Δ Steric hindrance by mutation | 0.0164 | 0.0529 |
| Standard | Δ P(sheet) by mutation | 0.0478 | 1.72E-08 |
| ND | Distance from ATP | 0.4319 | 4.60E-29 |
| ND | SASA by position | 0.3814 | 4.47E-22 |
| ND | ΔΔG by mutation | -0.1813 | 8.25E-89 |
| ND | Δ Hydrophobicity by mutation | 0.1294 | 1.00E-52 |
| ND | Δ Flexibility by mutation | 0.0980 | 6.80E-31 |
| ND | Δ pI by mutation | -0.0601 | 1.47E-12 |
| ND | Δ log(Solubility) by mutation | -0.0394 | 3.47E-06 |
| ND | Δ Volume by mutation | -0.0156 | 0.0670 |
| ND | Δ P(helix by mutation) | -0.0322 | 0.0002 |
| ND | Δ Steric hindrance by mutation | 0.0288 | 0.0007 |
| ND | Δ P(sheet) by mutation | 0.0661 | 6.85E-15 |
| Salt | Distance from ATP | 0.4086 | 6.60E-26 |
| Salt | SASA by position | 0.3036 | 3.55E-14 |
| Salt | ΔΔG by mutation | -0.1391 | 6.31E-53 |
| Salt | Δ Hydrophobicity by mutation | 0.0744 | 1.48E-18 |
| Salt | Δ Flexibility by mutation | 0.0653 | 0.0653 |
| Salt | Δ pI by mutation | 0.0122 | 0.1490 |
| Salt | Δ log(Solubility) by mutation | 0.0164 | 0.0524 |
| Salt | Δ Volume by mutation | -0.0080 | 0.3443 |
| Salt | Δ P(helix by mutation) | -0.0246 | 0.0038 |
| Salt | Δ Steric hindrance by mutation | 0.0072 | 0.3976 |
| Salt | Δ P(sheet) by mutation | 0.0364 | 1.76E-05 |
| Ethanol | Distance from ATP | 0.3699 | 3.49E-21 |
| Ethanol | SASA by position | 0.3459 | 3.44E-18 |
| Ethanol | ΔΔG by mutation | -0.1200 | 8.08E-40 |
| Ethanol | Δ Hydrophobicity by mutation | 0.1014 | 3.73E-33 |
| Ethanol | Δ Flexibility by mutation | 0.0799 | 3.60E-21 |
| Ethanol | Δ pI by mutation | -0.0564 | 2.67E-11 |
| Ethanol | Δ log(Solubility) by mutation | -0.0194 | 0.0218 |
| Ethanol | Δ Volume by mutation | -0.0293 | 0.0005 |
| Ethanol | Δ P(helix by mutation) | -0.0178 | 0.0352 |
| Ethanol | Δ Steric hindrance by mutation | 0.0216 | 0.0106 |
| Ethanol | Δ P(sheet) by mutation | 0.0465 | 4.04E-08 |
| Diamide | Distance from ATP | 0.3251 | 1.86E-16 |
| Diamide | SASA by position | 0.3666 | 2.16E-20 |
| Diamide | ΔΔG by mutation | -0.1536 | 3.33E-64 |
| Diamide | Δ Hydrophobicity by mutation | 0.1026 | 7.41E-34 |
| Diamide | Δ Flexibility by mutation | 0.0891 | 6.44E-26 |
| Diamide | Δ pI by mutation | -0.0475 | 2.08E-08 |
| Diamide | Δ log(Solubility) by mutation | -0.0144 | 0.0889 |
| Diamide | Δ Volume by mutation | -0.0213 | 0.0121 |
| Diamide | Δ P(helix by mutation) | -0.0198 | 0.0193 |
| Diamide | Δ Steric hindrance by mutation | 0.0146 | 0.0855 |
| Diamide | Δ P(sheet) by mutation | 0.0414 | 1.02E-06 |
| 37°C | Distance from ATP | 0.3178 | 9.24E-16 |
| 37°C | SASA by position | 0.3527 | 6.78E-19 |
| 37°C | ΔΔG by mutation | -0.1869 | 3.06E-95 |
| 37°C | Δ Hydrophobicity by mutation | 0.1391 | 2.83E-61 |
| 37°C | Δ Flexibility by mutation | 0.1124 | 1.72E-40 |
| 37°C | Δ pI by mutation | -0.0411 | 1.19E-06 |
| 37°C | Δ log(Solubility) by mutation | 0.0040 | 0.6381 |
| 37°C | Δ Volume by mutation | -0.0428 | 4.10E-07 |
| 37°C | Δ P(helix by mutation) | -0.0276 | 0.0011 |
| 37°C | Δ Steric hindrance by mutation | 0.0160 | 0.0592 |
| 37°C | Δ P(sheet) by mutation | 0.0553 | 6.33E-11 |
|  |  |  |  |
|  |  |  |  |
|  |  |  |  |
